# Champions as co‐designers in cerebral palsy knowledge translation: Turning evidence into practice

**DOI:** 10.1111/dmcn.70092

**Published:** 2025-11-30

**Authors:** Egmar Longo

**Affiliations:** ^1^ Department of Physiotherapy Federal University of Paraiba João Pessoa Brazil

## Abstract

**This commentary is on the original article by Hanson et al. on pages** 358–380 **of this issue.**

What still holds us back from putting early rehabilitation into practice everywhere? We know that cerebral palsy (CP) can be detected early.^1^ We have clear guidelines, ‘green light’ interventions,^2^ and strong foundations like the F‐words for child development.^3^ And yet, implementation moves at very different speeds across contexts. Hanson et al. take on this challenge by testing a multifaceted knowledge translation strategy—co‐designed with site champions—to help clinicians move from evidence to action in early CP rehabilitation.^4^

Early rehabilitation is universally recognized as critical, but the gap between research and practice remains stubborn. Clinicians often know the evidence, yet time pressures, scarce resources, and established routines slow down change. Hanson et al. provide valuable insights by testing the Early Detection and Intervention Toolkit for Cerebral Palsy (EDIT‐CP) electronic knowledge translation toolkit within a knowledge translation strategy that combined online training, newsletters, and local champions. Crucially, this was not a top‐down initiative: the toolkit was co‐developed with clinicians, caregivers, and champions, making sure it reflected both research evidence and the realities of practice. When implementation is co‐designed with the public, the impact is stronger.

The results are encouraging. Professionals reported greater engagement in evidence‐based practice, better access to resources, and found the toolkit useful for guiding decisions, educating families, and even advocating for organizational support. The participatory design clearly added credibility and trust. Still, challenges remain. Attitudes and confidence toward evidence‐based practices shifted little, and about a third of participants expressed limited intention to keep using the toolkit beyond the study. Some felt it was too focused on technical skills and not easily adaptable to functional, family‐centered goals that are prioritized in rehabilitation practice. This reminds us that toolkits and training are necessary, but not enough without broader organizational and cultural change.

The role of champions deserves attention. In this study, they were not only facilitators but also co‐creators of the knowledge translation strategy—bridging research and practice, and giving it credibility. But their positions depended on project funding, which raises questions about sustainability. Unless institutions formally recognize and protect these roles, especially in low‐ and middle‐income countries such as Brazil where patient and public involvement is still being established, progress may be slow.^5^

The implications are clear. For clinicians, digital knowledge translation tools like EDIT‐CP can accelerate decisions and strengthen communication with families, but organizational support is needed to sustain them. For healthcare systems, participatory co‐design should become the standard, ensuring knowledge translation initiatives reflect the voices of those delivering and receiving care. For research, we need long‐term studies that test sustainability, adaptations across health systems, and whether strategies like EDIT‐CP ultimately improve outcomes for children with CP. Communities of practice, interprofessional learning, and embedding knowledge translation tools into training programs may further support this process.

In the end, the study by Hanson et al. shows that multifaceted knowledge translation strategies can support evidence use in early CP rehabilitation, especially when champions are central to design and delivery. But evidence alone will never be enough. Real change depends on people, context, and systems. Sustaining that change means recognizing that champions—and the lived voices they bring into practice—are not optional, but vital agents of progress.

## FUNDING INFORMATION

The author has nothing to report.

## CONFLICT OF INTEREST STATEMENT

The authors declare no conflict of interest.

## Data Availability

Not required.
